# Editorial: The Role of Gene Polymorphisms in Modulating the Immune Responses Against Tropical Infectious Diseases

**DOI:** 10.3389/fimmu.2021.714237

**Published:** 2021-07-22

**Authors:** Adriana Malheiro, Rajendranath Ramasawmy, David Courtin, Eduardo Antonio Donadi

**Affiliations:** ^1^ Departamento de Parasitologia, Programa de Pós-graduação em Imunologia Básica e Aplicada, Universidade Federal do Amazonas, Manaus, Brazil; ^2^ Fundação Hospitalar de Hematologia e Hemoterapia do Amazonas, Laboratório de Genômica-Rede Regesam, Manaus, Brazil; ^3^ Faculdade de Medicina, Universidade Nilton Lins, Manaus, Brazil; ^4^ Fundacão de Medicina Tropical-Doutor Heitor Vieira Dourado, Manaus, Brazil; ^5^ UMR 261 MERIT, Université de Paris, Institut de Recherche pour le Développement (IRD), Paris, France; ^6^ Division of Clinical Immunology, Department of Medicine, Ribeirão Preto Medical School, University of São Paulo (USP), Ribeirão Preto, Brazil

**Keywords:** infectious diseases, HLA, immune response, gene polymorphism, tropical diseases

Immune responses against pathogens can be modulated by T and B regulatory cells, surface-bound and soluble immune checkpoint molecules, neutralizing antibodies and antibody receptors, soluble mediators produced during the immune response, and intracellular molecules, among others. Immune checkpoint molecules may promiscuously inhibit several types of cells of the immune system. For example, HLA-G may inhibit the function of T, B, natural killer, and antigen presenting, cells upon ligation with specific receptors on immune cells. The description of the in *silico* structures of the membrane-bound and of the soluble HLA-G1 and G5 isoforms (shown on the cover page and in the related article) is important to understand the docking of HLA-G with its major receptors (Arns et al.). Other immune checkpoint molecules may specifically inhibit a single or a group of lymphocyte subsets after cell activation, including programmed cell death protein-1 (PD-1). Immune responses against pathogens may be further modulated by the presence of nucleotide variation in genes responsible for the immune responses. In this Research Topic, nucleotide variants in genes associated with the control of the adaptive and innate immune responses are reported in viral, bacterial, fungal, and protozoan infections.

Interaction of PD-1 (*PDCD1* gene) with its ligands (PD-L1 and PD-L2) inhibits cellular immune responses and maintains the balance between activation and tolerance. The study of the *PDCD1* -606G>A (rs36084323) promoter region polymorphism in cervical lesions caused by high-risk human papillomavirus revealed that the mutant -606A allele is associated with increased protein and gene expression, irrespective of lesion severity. Additionally, a differential *PDCD1* expression was observed according to the severity of the lesion, indicating that the virus may escape immune control by regulating PD1 (da Silva et al.).

Cytophilic IgG antibodies bind to Fc gamma receptors (FcgR) expressed at the surface of immune cells, and genetic variability at the IgG heavy chain constant region and in FcgR may modulate the susceptibility to malaria infections. The study of the variability in the *FcgR* (*FcgRIIA*, *FcgRIIIA*, *FcgRIIIB NA1/NA2*) genes in Beninese children revealed that carriers the G3m24 single allotype and the G3m5,6,10,11,13,14,24 phenotype were independently associated with risk for malaria infection. In addition, several combinations of the *FcgRIIA, FcgRIIIA*, and *FcgRIIIB* polymorphisms and the IgG G3m allotypes were also associated with susceptibility to malaria, particularly in conditions of high intensity of exposure to mosquito bites, emphasizing the importance of cytophilic IgG1 and IgG3 isotypes in protection against *Plasmodium falciparum* malaria (Fall et al.).

Cytokine/chemokine gene diversity was evaluated in Chagas disease, paracoccidioidomycosis, leprosy, and tuberculosis. In Brazilian Chagasic patients, 35 Tag single nucleotide polymorphisms along the *IL12B*, *IL10*, *IFNG* and *IL4* cytokine genes were evaluated in patients exhibiting chronic cardiomyopathy and in asymptomatic patients. Only, the *IL12* (rs2546893G, rs919766C) and *IL10* (rs3024496C) alleles were associated with an increased risk for cardiomyopathy development (Frade-Barros et al.). Additionally, the promoter region *IL6* −174CG (rs1800795) genotype was associated with decreased risk for *Trypanosoma cruzi* detection whereas the *IL17A* −152AA (rs2275913), *IL18* −607AA (rs1946518), *IL1B* -31TC (rs1143627) genotypes and the HIV co-infection were associated with reduced risk for developing cardiomyopathy or severe cardiac dysfunction (dos Santos et al.). In Brazilian paracoccidiodomycosis patients, evaluation of cytokine and cytokine receptor genes (*IL12A, IL18* and *IFNGR1*) revealed that the *IL18* -607A allele was associated with susceptibility for the acute and chronic multifocal forms, whereas the *IL18* -607C allele was associated with protection against the unifocal chronic form (Sato et al.). The *IL10* polymorphism were studied in Indian patients presenting lepromatous leprosy. The promoter region *IL10* -819TT (rs1800871) and -1082GG (rs1800896) genotypes were overrepresented in patients, and the -819TT genotype was associated with an increased number of IL-10-producing CD4 and Treg cells, and increased skin expression of IL-10, supporting a significant role for IL-10 in disease pathogenesis (Tarique et al.).

Eight variants located in the promoter region of the chemokine receptor *CCR5* gene were investigated in Han Chinese patients infected with *Mycobacterium tuberculosis* (Mtb*)*. The rs2734648-G at single or double doses and the rs1799987-AA genotype were associated with susceptibility to tuberculosis or pulmonary tuberculosis. Haplotype analysis of the eight variants revealed that the CCR5 rs2227010A-rs2856758A-rs2734648G-rs1799987G-rs1799988T-rs41469351C-rs1800023G-rs1800024C haplotype increased susceptibility to tuberculosis and pulmonary tuberculosis (Liu et al.). This study sheds light on the importance of CCR5 in the regulation, recruitment, and activation of macrophages. An increase in the expression of CCR5 might be detrimental and lead to the development of TB.

Considering that host innate immune genes may shape susceptibility to pathogens, inflammasome gene polymorphisms (nucleotide-binding oligomerization domain-like receptor pyrin domain containing-3 (*NLRP3)* C>G rs10754558, nucleotide-binding oligomerization domain-like receptor-CARD domain-containing protein 4 (*NLRC4)* G>C rs479333, absent in 28 melanoma 2 *(AIM2)* T>C rs1103577, Caspase-Activation and Recruitment Domain-8 (*CARD8*) T>C rs2009373, and cathepsin B (*CTSB)* A>C rs1692816) were evaluated in tuberculosis in two Brazilian studies. The gain-of-function *NLRP3* GG (rs10754558) genotype was associated with increased IL-1ß production when stimulated with Mtb compared to non-carriers, representing an important sensor for Mtb virulence, whereas the presence of at least one lost-of-function *NLRC4* C (rs479333) allele was associated with extra-pulmonary tuberculosis (De Lima et al.). The *AIM2* CC (rs1103577) genotype was associated with increased risk for pulmonary tuberculosis development, since patients carrying this genotype exhibited high levels of IL-1β. The *CTSB* gene (rs1692816) was associated with reduced risk for extra-pulmonary manifestations (Figueira et al.). These studies highlight the importance of inflammasomes in the development of TB and suggest that individuals predisposed to high levels of IL-1β production were more susceptible to the development of TB.

An innate immune retroviral restriction factor was also studied. The Sterile Alpha Motif (SAM) and Histidine- Aspartic (HD) domain-containing protein 1 (*SAMHD1)* gene is a dNTPase that prevents the synthesis of double-stranded DNA, impairing viral replication cycle and viral integration into the cell genome. The G allele of the *SAMHD1* A>G (rs6029941) 3’ untranslated region (3’UTR) polymorphism was associated with a higher pro-viral load and lower levels of IFN-α in human T-cell lymphotropic virus type 1 (HTLV1) infected patients from the Brazilian Amazon area, suggesting that microRNAs may differentially target this polymorphic site (Queiroz et al.).

This study identifies individual that carry the G allele and produce low levels of IFN-α as having higher proviral load during HTLV1 infection. Furthermore, a possible influence of microRNA acting in this region may regulate the expression of the gene.

Finally, a review article, focusing on the role of genetic factors in schistosomiasis, identified several major quantitative trait *loci* (QTL), including *Schistosoma mansoni*, susceptibility/resistance region 1 (SM1) located in the region (5q31-q33) that are associated with parasite burden. This region harbors several genes of the Th2 immune response. A second QTL (SM2, 6q22-q23) was associated with the intensity of liver fibrosis. QTLs interspersed in SM1 and SM2 regions, were related to genes of the Th17 profile (Mewamba et al.). This review demonstrates the importance of the host genetic background in the controlling of the parasite.

Altogether, this series of papers describe the differential impact of nucleotide variation in genes related to the control of the innate and/or adaptive immune response against infectious diseases. Variations observed in exonic regions may modify protein function, whereas polymorphisms observed at the regulatory regions (promoter and 3’UTR) may impact gene expression. Accordingly, this series provides evidence that nucleotide variation in genes associated with the control of the immune response against tropical infectious diseases impacts pathogen escape from the specific immune response, disease pathogenesis, and disease morbidity.

## Author Contributions

All authors contributed to the article and approved the submitted version. For the elaboration of the editorial paper, all contributed with the writing and revisions.

## Conflict of Interest

The authors declare that the research was conducted in the absence of any commercial or financial relationships that could be construed as a potential conflict of interest.

